# Exome sequencing of hepatocellular carcinoma in lemurs identifies potential cancer drivers: A pilot study

**DOI:** 10.1093/emph/eoac016

**Published:** 2022-04-29

**Authors:** Ella F Gunady, Kathryn E Ware, Sarah Hoskinson Plumlee, Nicolas Devos, David Corcoran, Joseph Prinz, Hrvoje Misetic, Francesca D Ciccarelli, Tara M Harrison, Jeffrey L Thorne, Robert Schopler, Jeffrey I Everitt, William C Eward, Jason A Somarelli

**Affiliations:** 1 Department of Medicine, Duke University Medical Center, Durham, NC 27710, USA; 2 Department of Orthopaedics, Duke University Medical Center, Durham, NC 27710, USA; 3 Duke Center for Genomic and Computational Biology, Duke University Medical Center, Durham, NC 27710, USA; 4 Cancer Systems Biology Laboratory, The Francis Crick Institute, London NW1 1AT, UK; 5 School of Cancer and Pharmaceutical Sciences, King’s College London, London SE1 1UL, UK; 6 Department of Clinical Sciences, North Carolina State University, College of Veterinary Medicine, Raleigh, NC, USA; 7 Exotic Species Cancer Research Alliance, College of Veterinary Medicine, North Carolina State University, Raleigh, NC, USA; 8 Department of Biological Sciences, North Carolina State University, Raleigh, NC, USA; 9 Department of Statistics, North Carolina State University, Raleigh, NC, USA; 10 Duke Lemur Center, Durham, NC 27705, USA; 11 Department of Pathology, Duke University Medical Center, Durham, NC 27710, USA; 12 Duke Cancer Institute, Durham, NC 27710, USA

**Keywords:** liver cancer, mutation, prosimians, non-human primates, TP53, ARID1A, CTNNB1

## Abstract

**Background and objectives:**

Hepatocellular carcinoma occurs frequently in prosimians, but the cause of these liver cancers in this group is unknown. Characterizing the genetic changes associated with hepatocellular carcinoma in prosimians may point to possible causes, treatments and methods of prevention, aiding conservation efforts that are particularly crucial to the survival of endangered lemurs. Although genomic studies of cancer in non-human primates have been hampered by a lack of tools, recent studies have demonstrated the efficacy of using human exome capture reagents across primates.

**Methodology:**

In this proof-of-principle study, we applied human exome capture reagents to tumor–normal pairs from five lemurs with hepatocellular carcinoma to characterize the mutational landscape of this disease in lemurs.

**Results:**

Several genes implicated in human hepatocellular carcinoma, including *ARID1A*, *TP53* and *CTNNB1*, were mutated in multiple lemurs, and analysis of cancer driver genes mutated in these samples identified enrichment of genes involved with *TP53* degradation and regulation. In addition to these similarities with human hepatocellular carcinoma, we also noted unique features, including six genes that contain mutations in all five lemurs. Interestingly, these genes are infrequently mutated in human hepatocellular carcinoma, suggesting potential differences in the etiology and/or progression of this cancer in lemurs and humans.

**Conclusions and implications:**

Collectively, this pilot study suggests that human exome capture reagents are a promising tool for genomic studies of cancer in lemurs and other non-human primates.

**Lay Summary:**

Hepatocellular carcinoma occurs frequently in prosimians, but the cause of these liver cancers is unknown. In this proof-of-principle study, we applied human DNA sequencing tools to tumor–normal pairs from five lemurs with hepatocellular carcinoma and compared the lemur mutation profiles to those of human hepatocellular carcinomas.

## INTRODUCTION

Endemic to Madagascar, lemurs are a diverse group of primates comprised of five families and dozens of species [1]. According to the International Union for Conservation of Nature Red List, 31% of all lemur species are critically endangered and are at high risk of becoming extinct [2]. Declines in lemur populations are primarily driven by habitat destruction and illegal hunting [2]. Both local and international conservation efforts are attempting to counter these burdens on population recovery. Critical to these conservation efforts are research programs to better understand the species, its natural environment, and common diseases and conditions [3].

Among the pathologies common to lemurs, hepatocellular carcinoma is the most common spontaneous neoplasm in prosimians, the primate group encompassing all lemur species [4]. A histological study of adults in a managed population of prosimians found a relatively high rate of metastasis in prosimians with hepatocellular carcinoma [5]. The underlying causes of hepatocellular carcinoma in prosimians remain unclear. Proposed risk factors of the disease in lemurs include excess iron deposition (hemosiderosis/hemochromatosis), hepatitis virus infection, cobalt deficiency and dietary aflatoxin B1 (AFB1) exposure [5]. While the genetic changes associated with these etiologies in lemurs have not been well-studied, several of these etiologies have been associated with specific genes and mutations in humans. For example, a G>T transversion in the third position of codon 249 of the *TP53* gene has been pinpointed as a mutational hot spot in hepatocarcinogenesis frequently associated with AFB1 exposure [6]. Additionally, the hepatitis B virus (HBV) X gene, which is frequently integrated into the chromosomal DNA of patients with HBV-induced hepatocellular carcinoma, encodes a multifunctional protein that modulates DNA repair, cell cycle progression and p53-mediated apoptosis [6]. Uncovering the genetic changes associated with hepatocellular carcinoma in lemurs may thus point to possible causes of the disease in these animals and, consequently, suggest potential therapeutic targets and treatments or changes in regimens at conservation sites.

Comparison of cancer across species can illuminate fundamental drivers of cancer initiation and progression [7]. The spontaneous occurrence of cancer in lemurs presents a valuable opportunity to identify similarities and differences in cancer mutation profiles among closely-related species. While there is a critical need to better understand the underlying genomic features of cancers in non-human primates, particularly in lemurs, genomic studies of cancer mutation profiles in these animals have been hampered by a lack of tools with which to perform these studies. The recent observation that human exome capture reagents can be used across primates provides a potential avenue to explore the mutational landscapes of cancers in lemurs and other closely-related species [8, 9]. In this study, we attempted to test if human exome reagents and tools could be applied to analysis of hepatocellular carcinoma in lemurs. Applying this platform to five tumor–normal pairs of lemur hepatocellular carcinoma, we found that hepatocellular carcinoma from the five lemurs studied shares several notable characteristics with human hepatocellular carcinoma, including mutations in the tumor suppressors *ARID1A* and *TP53* and the oncogene *CTNNB1*; analysis of cancer driver genes mutated in these samples identified enrichment of genes involved with *TP53* degradation and regulation.

## MATERIALS AND METHODS

### Whole-exome sequencing and identification of variants

Tumor–normal pairs were selected from banked flash frozen tissue from the Duke Lemur Center. Pathologic evaluation of hematoxylin and eosin-stained slides was used to confirm hepatocellular carcinoma in the tumor samples, estimate tumor content and confirm the absence of tumor in the paired normal samples. Total genomic DNA was isolated using the Quick-DNA Miniprep kit (Zymo Research, 11-317C). For exome sequencing, extracted DNA was quantified using Qubit (Thermo Fisher Scientific). DNA-seq libraries were prepared for each sample using the KAPA HyperPrep kit (Roche). Final libraries were quality checked using Qubit and Bioanalyzer (Agilent) and pooled in batches of 12 (pre-capture pooling). Each pool of 12 libraries was then hybridized with IDT Human xGen Exome Research Panel V1 probes in order to capture and pull down the portion of the DNA-seq library representative of the lemur exome. Final captured libraries were amplified, pooled and sequenced on one lane of an Illumina NovaSeq 6000 S-Prime flow cell. Sequencing was done at 150 bp PE. Sequence data were demultiplexed, and Fastq files generated using Illumina bcl2fastq conversion software.

Exome-seq data was processed using fastp [10] to trim low-quality bases and Illumina sequencing adapters from 3′ end of the reads. Reads were aligned to the Mouse Lemur genome (Mmur3, ftp://ftp.ensembl.org/pub/release-100/fasta/microcebus_murinus/dna/) using BWA [11] algorithm and PCR duplicates were flagged using PICARD Tools [12] software suite. Subsequent alignment processing and variant calling were performed on the matched-normal samples using the GATK [13] recommended workflow for detecting somatic variants using Mutect2 [14]. Variants in the VCF files were filtered for passing variants with a sum of allele depth values greater than seven and annotated using the Ensembl Variant Effect Predictor [15], with reference genome Mmur_3.0. All data have been deposited to the European Variation Archive (project accession number PRJEB51774).

### Mutational profile analysis

The BSgenome R package [16] was used to forge a package containing the genome for *Microcebus murinus* (Mmur3). The mutSignatures R package [17] was used to count the number of single-nucleotide variants in each trinucleotide context, using Mmur3 as the reference genome. The deconstructSigs R package [18] was used to determine the relative contribution of each COSMIC (Catalogue of Somatic Mutations in Cancer v3.2) [19] single base substitution signature to the total substitution burden of each tumor. The human hepatocellular carcinoma mutational profile was obtained from MutaGene data and was constructed from over 1,000 samples of human hepatocellular carcinoma [20].

### Identification of cancer driver genes

All mutated genes in the five lemurs were intersected with a list of 16, 656 lemur genes with human orthologs from bioDBnet [21] and annotated using Variant Effect Predictor [15]. This resulted in 12, 448 lemur genes mutated 54, 868 times, which were used as input for sysSVM2 [22]. Only exonic and splicing mutations were kept. Frameshift, stop-gain and stop-loss and non-synonymous mutations were considered as potentially damaging. Systems-level properties were retrieved as previously described [22]. SysSVM2 was trained on 236 known cancer genes derived from the Network of Cancer Genes (NCG) [23] with damaging mutations in 283 hepatocellular carcinoma samples from The Cancer Genome Atlas (TCGA). Model parameters for each kernel (linear, polynomial, radial, sigmoid) were assessed through 3-fold cross-validation with 10, 000 iterations as previously described [22]. A score was assigned to each mutated gene, with a higher score representing higher similarity to the features of known cancer genes. Cancer driver genes in each lemur were defined, prioritizing mutated hepatocellular carcinoma driver genes [23] and then adding the highest-ranking sysSVM2 predictions until reaching eight driver genes per sample. This number was derived from the literature [24].

### Pathway enrichment analysis on cancer driver genes

Pathway enrichment analysis was conducted using 1,303 biological pathways from Reactome [25] (v72) composed of 10–500 genes. A total of 41 unique driver genes were mapped to 337 pathways and enrichment was assessed using a one-sided hypergeometric test. A total of 45 pathways with a false discovery rate (Benjamini–Hochberg correction) below 0.05 were considered enriched (Supplementary Table S3).

### Mutated genes and pathways and comparison with human hepatocellular carcinoma

Over-representation analysis was performed using g:Profiler’s g:GOSt tool [26] (version e103_eg50_p15_68c0e33) with the g: SCS multiple testing correction method applying a significance threshold of 0.05. The organism was set to human (version GRCh38.p13), and the following gene sets were considered: GO molecular function (01 February 2021 release), GO cellular component (01 February 2021 release), GO biological process (01 February 2021 release) [27] and WikiPathways [28] (10 March 2021 release). Genes mutated in lemur hepatocellular carcinoma were compared to those mutated in human hepatocellular carcinoma within five studies compiled in cBioPortal [29–33]. Mutations shared across lemurs were identified and visualized using the VennDiagram and ComplexHeatmap R packages [34, 35].

## RESULTS

### Whole-exome sequencing of lemur hepatocellular carcinomas

**Figure 1.**
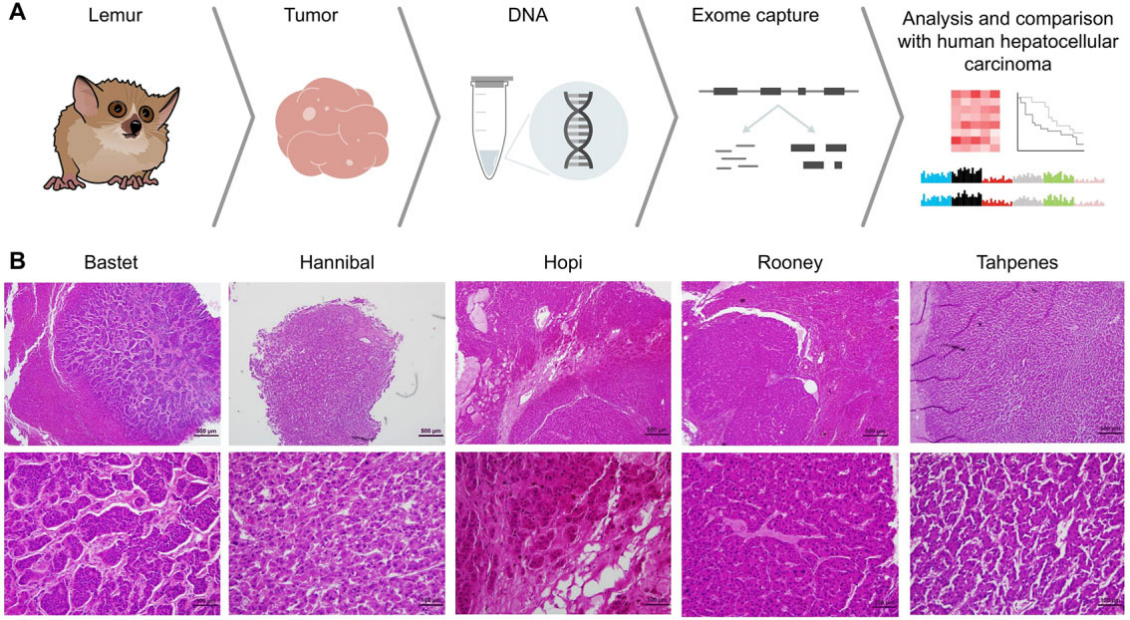
Analysis of hepatocellular carcinoma in lemurs. (**A**) Workflow of the isolation and analysis of DNA from lemurs with hepatocellular carcinoma. (**B**) Low magnification (40×, top row) and high magnification (200×, bottom row) photomicrographs of tumors from each lemur, whose names are listed as column headers

To identify mutations observed in lemur hepatocellular carcinoma, we performed whole-exome sequencing on five tumor–normal pairs from lemurs with human capture reagents (see Table 1 for species list). Tumor–normal pairs were selected from banked flash frozen tissue of lemurs with hepatocellular carcinoma (Fig. 1). Histopathology evaluation confirmed samples as hepatocellular carcinoma, and the proportion of neoplastic content versus normal hepatic parenchyma was estimated. Hepatocellular carcinoma was observed in 50%, 100%, 80%, 50% and 80% of the tumor tissue sections from Bastet, Hannibal, Hopi, Rooney and Tahpenes, respectively.

**Table 1.** Overview of variants identified in whole-exome sequencing data

Name of lemur	Sex	Age (years)	Species	Total number of mutations	Number of high impact mutations
Bastet	F	26	*Eulemur coronatus*	878	22
Hannibal	M	29	*Eulemur* hybrid (*sanfordi* and *rubriventer*)	51, 444	322
Hopi	F	25	*Eulemur rubriventer*	2,130	52
Rooney	M	18	*Eulemur flavifrons*	1,377	40
Tahpenes	F	27	*Eulemur albifrons* father, *albifrons* hybrid mother	973	32

**Figure 2.**
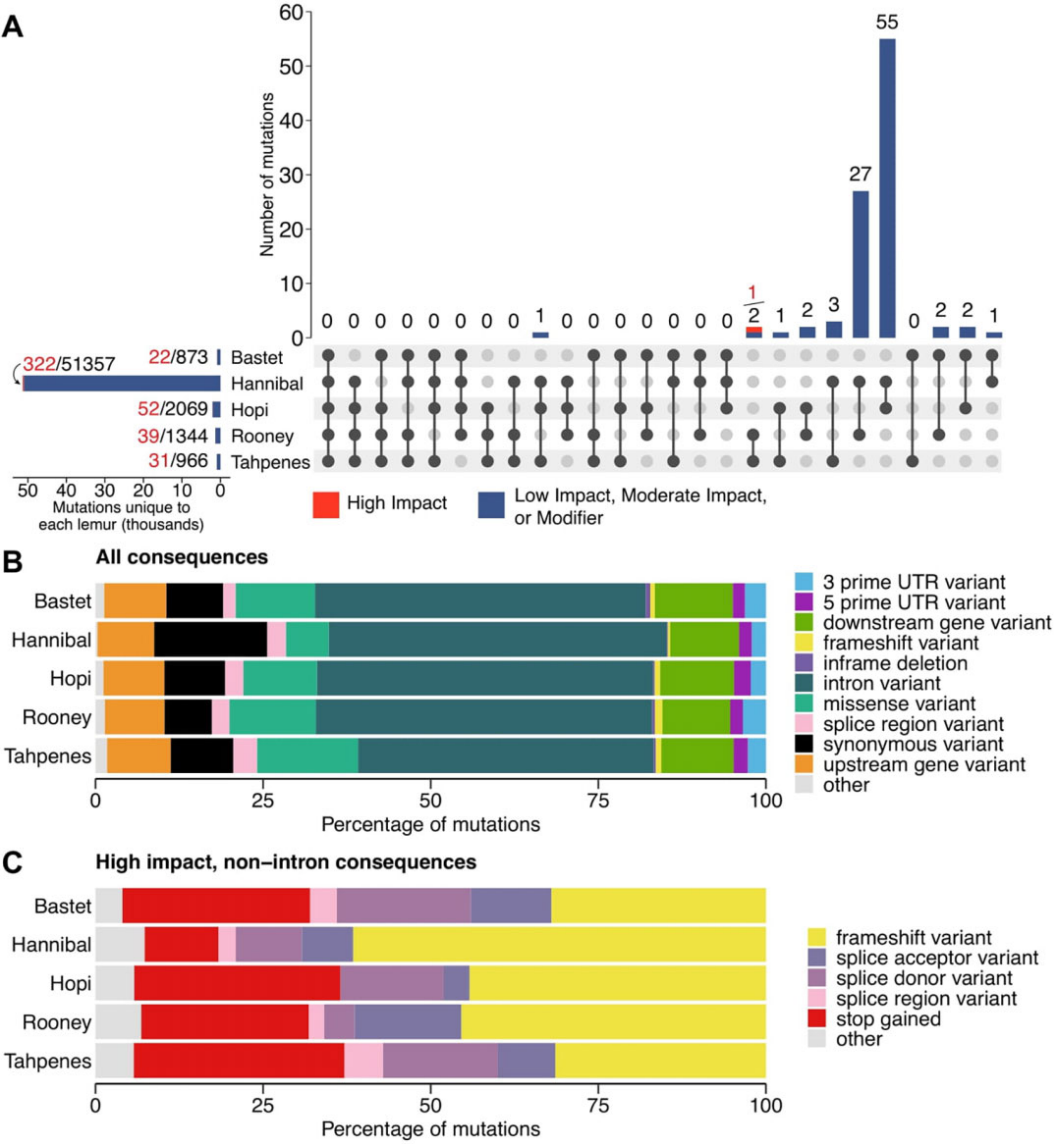
Comparison of exome mutations across lemurs. (**A**) Number of mutations unique to (horizontal bars) or shared across (vertical bars) lemurs, where mutations are considered unique based on location. The dots and lines show which lemurs are included in the overlap. Mutation numbers are summarized as #/#, where the numbers to the left indicate number of high impact mutations, and numbers on right indicate total number of mutations. Venn diagrams showing these overlaps can be found in Supplemental Figure 2. (**B, C**) Overview of exome mutations’ consequences as predicted by Variant Effect Predictor

Using human exome capture reagents, we captured a total of 78.45% (45 Mb) of the lemur exome in at least a single sample with an average coverage of 124×. A total of 40.67% (24 Mb) of the lemur exome was captured with an average depth of 25× across all the samples (Supplementary Fig. S1 and Table S1). Unique mutations were counted based on chromosome location, and intersections across lemurs were visualized as an UpSet plot [36] (Fig. 2A). While the majority of mutations are heterogeneous in chromosome location, Hannibal, Hopi and Tahpenes each had an in frame deletion at the same chromosome position (11:96169749–96169752) in the gene HECW1. Rooney and Tahpenes each had a single high impact nonsense mutation at the position 19:68353624–68353624 (Ensembl gene ID ENSMICG00000036843).

Not surprisingly, the largest percentage of variants was in introns, comprising an average of 48.8% of mutations. Missense and synonymous variants comprised an average of 11.4% and 10.2% of mutations, respectively. Frameshift variants and in-frame deletions made up an average of 0.74% and 0.34% of mutations, respectively (Fig. 2B). Among high impact variants, a mean of 42.9% were frameshift variants, and an average of 25.2% resulted in premature stop codons. Mutations categorized as splice acceptor, splice donor, or splice region variants comprised an average of 9.6%, 13.4% and 2.9% of high impact mutations, respectively (Fig. 2C). Percentages were calculated as the number of each type of variant divided by the total number of mutations, where this total considered variants of different consequences in the same chromosome location separately.

### Mutational profiles of lemur and human hepatocellular carcinoma

Mutational landscapes in cancers are shaped by mutational processes that can be distinguished by their distinct genetic signatures. This enabled the establishment of the COSMIC signatures, a compendium of mutational signatures found across the spectrum of human cancers.

**Figure 3.**
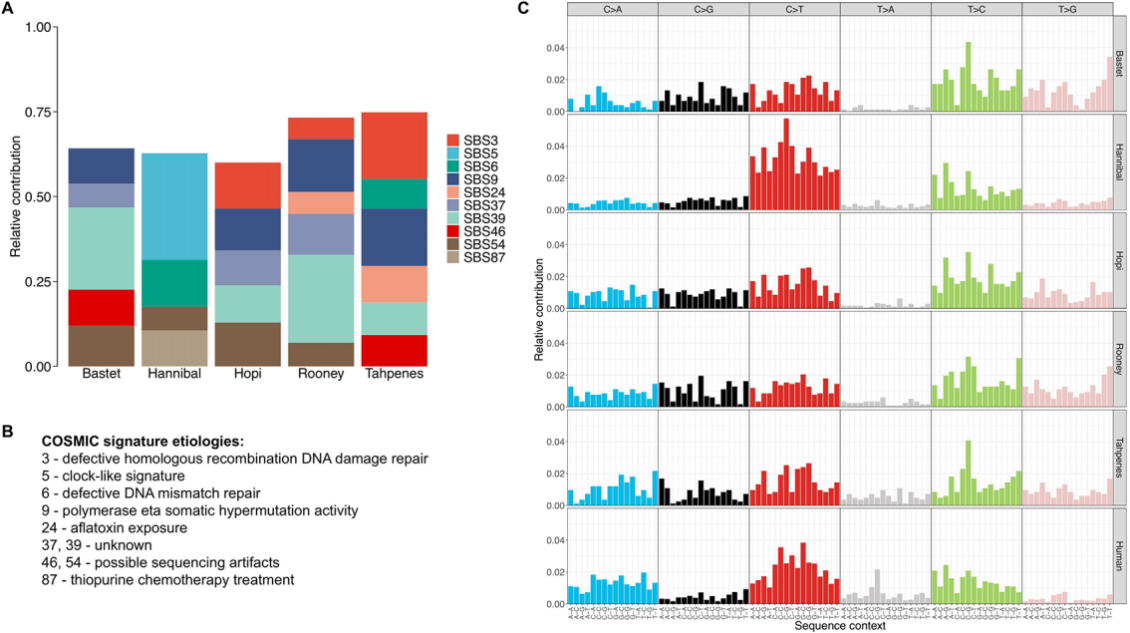
Comparison of mutational profiles in lemur and human hepatocellular carcinoma. (**A**) Relative contribution of COSMIC signatures to the mutational profiles in each lemur sample. The mutational profiles in these samples cannot be fully reconstructed from COSMIC signatures, which is why each bar does not sum to one. (**B**) COSMIC signature etiologies [19]. (**C**) Mutational profiles of hepatocellular carcinoma in lemur samples (first five rows) compared to humans (last row)

We used deconstructSigs to determine the relative contribution of each COSMIC single base substitution (SBS) signature to the mutational profile of each lemur. DeconstructSigs identified three signatures that contribute to four of the five lemurs’ mutational profiles: SBS9, which is associated with polymerase eta somatic hypermutation; SBS39, whose etiology is unknown; and SBS54, which is a possible sequencing artifact (Fig. 3A) [19]. SBS5, which has been found to correlate with age in several human cancer types, and SBS6, which is associated with defective mismatch repair and is found in microsatellite unstable tumors, contribute to 31.4% and 13.8% of the mutational profile of Hannibal, who has more than 20 times the number of mutations than the other lemurs [19, 37].

Given the limited sample size available for this study, we cannot draw broad conclusions about the mutational signatures of hepatocellular carcinoma in lemurs. However, plotting the mutational profiles of the individual lemurs revealed similarities across the lemurs, particularly in the relative absence of T>A mutations (Fig. 3C). Hannibal’s mutational profile is characterized by a notable bias in C>T mutations that is not apparent in the other lemurs, but is observable in human hepatocellular carcinomas.

### Cancer driver analysis pinpoints pathways related to TP53 regulation and degradation across all five lemurs

**Figure 4.**
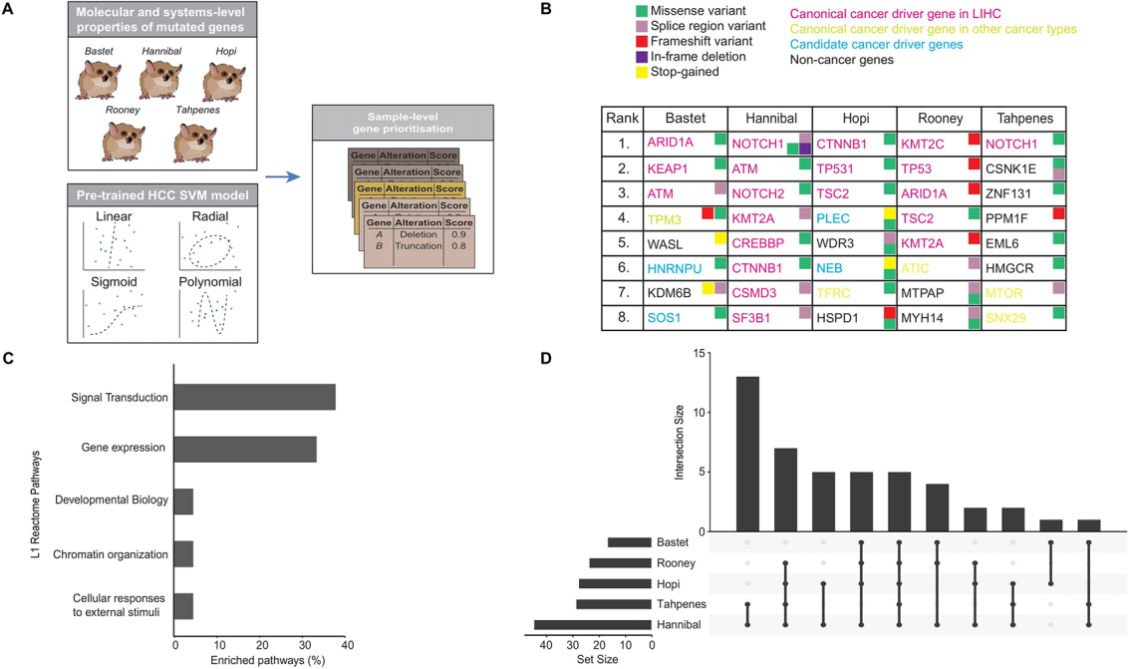
Identification of cancer driver genes in lemur hepatocellular carcinoma. (**A**) Modified workflow of sysSVM2 used to identify cancer driver genes (**B**) List of eight cancer driver genes (human orthologs) identified in each lemur sample with their variant annotation and cancer driver role in human cancers retrieved from NCG. (**C**) Proportion of pathways that are enriched in cancer driver genes after their mapping to top-level processes in Reactome. Showing level one pathways with at least two enriched sub-pathways. (**D**) Intersection of enriched pathways among all five lemur samples

We next applied a modified version of the sysSVM2 algorithm [22] for sample-specific identification of cancer driver genes. Molecular properties of mutated genes in lemurs were obtained from their variant annotation, while systems-level properties were retrieved from their corresponding human orthologs. This enabled us to apply a support vector machine that was trained on the properties of known cancer genes of human hepatocellular carcinoma to rank mutated genes with the most similar properties in each lemur (Fig. 4A).

All five lemurs have a mutation in at least one known human hepatocellular carcinoma driver gene while some sysSVM2 predictions are drivers in other human cancer types, suggesting that this methodology may be able to identify candidate drivers even in non-human primates (Fig. 4B). Hannibal has at least one substitution within 16 known human hepatocellular carcinoma driver genes. Most of Hannibal’s mutations are likely a consequence of mismatch repair deficiency, given that Hannibal has missense variants in *MLH1* and *MSH6* genes, and/or clock-like mutational processes (Fig. 3A). The mutation in DNA repair machinery is also consistent with this individual having 20 times more mutations than the other lemurs. Out of 41 unique cancer driver genes, only eight have mutations in two samples, but all mutations converged on perturbation of pathways related to signal transduction and gene expression (Fig. 4C, Supplementary Table S3). Five enriched pathways are common to all five samples, and three of them are involved in p53 degradation and regulation (Fig. 4D, Supplementary Table S4). These results suggest that individual-level driver mutations may cause perturbation of common core processes.

### Over-representation analysis identifies gene sets significantly represented in mutated lemur genes

**Figure 5.**
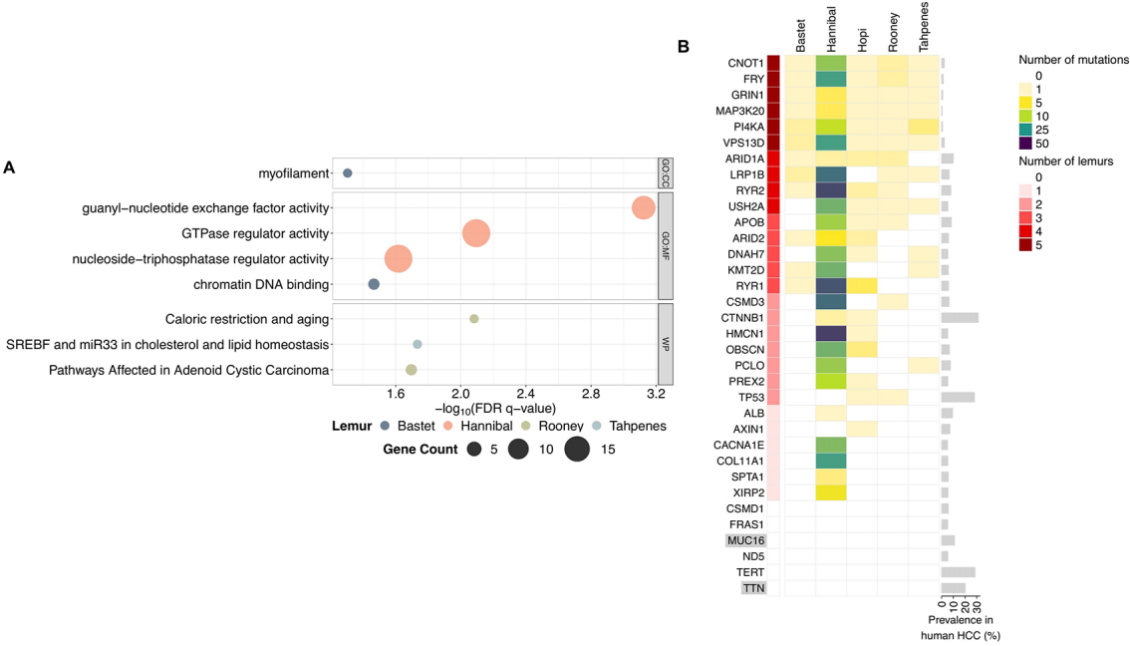
Over-representation analysis and comparison of lemur and human hepatocellular carcinoma mutations. (**A**) Dot plots of GO cellular component, GO molecular function and WikiPathways gene sets that significantly overlap with the high impact mutations of each lemur. The size of each dot is proportional to the number of mutated genes in the gene set for each lemur. No significant results were returned for Hopi. (**B**) Heatmap showing number of mutations in genes mutated in all five lemurs or frequently mutated in human hepatocellular carcinomas. Upstream and downstream variants were removed to construct this heatmap. CSMD1, FRAS1, ND5 and TERT were covered by exome sequencing, while MUC16 and TTN were not

Over-representation analysis was used to identify gene sets that significantly overlap with the genes containing high impact mutations in each lemur (Fig. 5A). This analysis pinpointed significant overlap with myofilament and chromatin DNA binding gene sets in Bastet; guanyl-nucleotide exchange factor activity, GTPase regulator activity and nucleoside-triphosphatase regulator activity gene sets in Hannibal; caloric restriction and aging and pathways affected in adenoid cystic carcinoma gene sets in Rooney; and SREBF and miR33 in cholesterol and lipid homeostasis in Tahpenes. No significant results were returned for the genes containing high impact mutations in Hopi.

### Comparison of mutations in human and lemur hepatocellular carcinoma reveals both similarities and differences

While this pilot study was not powered to detect statistically-reliable differences in mutations across species, we noted several gene-level features of lemur hepatocellular carcinoma that are consistent with human hepatocellular carcinoma and others that are unique to lemurs. For example, several of the genes mutated in four of the five lemurs are mutated in a subset of human hepatocellular carcinomas (Fig. 5B). These genes include *ARID1A*, a tumor suppressor and chromatin regulator [38] and *LRP1B*, which encodes a low-density lipoprotein receptor-related protein that is frequently mutated in cancers [39]. *ARID1A* and *LRP1B* are mutated in 10% and 6.4% of human hepatocellular carcinomas within five studies compiled in cBioPortal [29–33]. In addition, the oncogene *CTNNB1* and the tumor suppressor *TP53*, which are mutated in 31% and 28% of human hepatocellular carcinomas, were each mutated in two lemurs [38]. Although multiple gene-level mutations were similar across lemur and human hepatocellular carcinoma, there were also distinct differences. For instance, several genes that are mutated in human hepatocellular carcinoma were not observed in this pilot analysis. These genes include the telomerase reverse transcriptase gene, *TERT* [38], *CSMD1*, a putative tumor suppressor that is frequently mutated in human hepatocellular carcinoma associated with HBV infection [40], *FRAS1* and the mitochondrial encoded gene *ND5*. *TERT*, *CSMD1*, *FRAS1* and *ND5* are mutated in 28.6%, 5.7%, 5.2% and 5.3% of human hepatocellular carcinomas. *MUC16* and *TTN*, which are mutated in 11.1% and 20.2% of human hepatocellular carcinomas, were not captured by exome sequencing.

A total of six genes contained mutations in all five lemurs; yet, all of these genes are mutated in less than 2.5% of human hepatocellular carcinoma (Fig. 5B). Genes mutated in all five lemurs include *PI4KA*, which is necessary for the propagation of the hepatitis C virus and whose overexpression is associated with poor prognosis in human hepatocellular carcinoma [41] and *CNOT1*, which, together with IGF2 mRNA-binding proteins, regulates expression of the liver cancer-associated lncRNA HULC [42].

## DISCUSSION

Given the endangered status of lemurs and the prevalence of hepatocellular carcinoma in prosimians, understanding the genetic underpinnings of this disease in lemurs may inform conservation efforts by revealing potential causes of and treatments for hepatocellular carcinoma in these animals. Recent studies demonstrating the efficacy of using human-based exome capture methods on non-human primates present a promising opportunity to study cancer across species using existing cost-effective sequencing tools.

This pilot study seeks to evaluate the potential of using human-based sequencing technologies to understand cancer in closely related species. Our findings suggest that: (i) human exome capture reagents provide a useful tool set to evaluate the genomic landscape of lemur hepatocellular carcinoma at low cost without the need to develop custom libraries and (ii) the mutational landscape of hepatocellular carcinoma in lemurs shares key similarities and differences with human hepatocellular carcinoma.

The mutational spectrum in human germlines is characterized by a high frequency of C>T and T>C transitions, particularly at CpG sites [43]. A similar pattern is observed in human hepatocellular carcinoma, with a notable excess of C>T mutations [20]. With the exception of Hannibal, C>T transitions were relatively infrequent in this group of lemur hepatocellular carcinomas. This, and the recent observation that C>T mutations at CpG sites are less frequent in the gray mouse lemur compared to other primate species, may point to a correlation between germline and somatic mutational patterns in lemur cancers [44].

Among the identified mutations in lemur hepatocellular carcinoma with similarity to human hepatocellular carcinoma, we observed mutations in the tumor suppressors *ARID1A* and *TP53* and the oncogene *CTNNB1* [38]; cancer driver genes in all five samples suggested perturbation of *TP53* regulation and degradation. Studies of human hepatocellular carcinoma have identified etiologies with potential links to mutations in these genes: *ARID1A* mutations are common in hepatocellular carcinoma related to alcohol intake, G>T mutations at codon 249 of *TP53* are common in regions with dietary AFB1 exposure and HBV infection, and *CTNNB1* mutations are common in HCV-related hepatocellular carcinomas [38]. Although a hepatitis screen was not performed in this population, future studies may consider hepatitis screening to determine whether hepatitis-related hepatocellular carcinoma in lemurs is characterized by particular mutations. In addition, the DNA mismatch repair genes *MLH1* and *MSH6* contained missense mutations in Hannibal, who had more than 20 times more mutations than the other lemurs. Besides Bastet, who had a mutation in an *MLH1* intron, none of the other lemurs had mutations in these mismatch repair genes. Further studies of cancer genetics in lemurs may uncover a prognostic or predictive role of mutated mismatch repair pathways in non-human primate cancers and reveal similarities and differences in the role of repair mechanisms across primate cancers. Additionally, three lemurs had an inframe deletion at the same chromosome position (11:96169749–96169752) in the gene *HECW1*, and two lemurs had a single high impact nonsense mutation at the position 19:68353624–68353624. In the same way that the third position of codon 249 of the *TP53* gene is a mutational hot spot in human hepatocarcinogenesis, further sequencing studies may uncover mutational hot spots in lemur hepatocellular carcinoma.

While another study found a relatively high incidence of mutations in the H-*ras* gene in lemurs with hepatocellular carcinoma [45], this gene, which encodes a GTPase, contained only modifier or low impact mutations in two lemurs, Bastet and Hannibal. However, over-representation analysis identified significant overlap between the genes containing high impact mutations in Hannibal and three gene sets involving GTPase regulation. Genes containing high impact mutations in Bastet significantly overlapped with myofilament and chromatin DNA binding gene sets. Among these genes are *TPM3*, which is frequently overexpressed in human hepatocellular carcinoma, and the histone demethylase *KDM6B*, both of which may be involved in the epithelial–mesenchymal transition in hepatocarcinogenesis [46, 47]. The Wikipathway gene set, SREBF and miR33 in cholesterol and lipid homeostasis, significantly overlapped with the genes containing high impact mutations in Tahpenes; studies have identified differential expression of the microRNA miR33 and pathways involving SREBF1 in human hepatocellular carcinoma [48, 49]. The gene sets over-represented in Tahpenes’s high impact mutations were caloric restriction and aging and pathways affected in adenoid cystic carcinoma. Further research with larger cohorts may help to uncover the role of these gene sets in the development of hepatocellular carcinoma in prosimians.

While there remains much to be learned about the molecular pathogenesis of hepatocellular carcinoma in lemurs, this study suggests that human exome sequencing platforms may provide a low-cost alternative to evaluate the mutational landscape of lemur cancers and uncover mutational signatures in this species. Human liver cancer is characterized by distinct mutational signatures, with five COSMIC signatures (1, 4, 5, 12 and 16) accounting for 97% of mutations in human hepatocellular carcinoma [50]. COSMIC signature 16, which is considered a hallmark of human liver cancer, is associated with male gender and alcohol and tobacco consumption [50]. Similar to the way human liver cancers are driven by distinct mutational signatures, the lemur hepatocellular carcinoma samples share similar mutational signatures; however, the signatures identified in the five individuals analyzed are different from those identified in the vast majority of human hepatocellular carcinomas. Mutational signatures in the lemurs included COSMIC SBS39, SBS54 and SBS9, the latter of which is associated with polymerase eta somatic hypermutation in lymphoid cells [19]. These are distinct from the mutational signatures characteristic of hepatocellular carcinoma in humans, suggesting that the disease may be driven by unique signatures in lemurs. In the same way that identifying mutational signatures of cancers in humans can pinpoint potential causes and treatments, this strategy may be applied to hepatocellular carcinoma in lemurs. For example, COSMIC SBS5, which contributes to Hannibal’s mutational profile, is associated with aging in several human cancer types; SBS24, which is associated with AFB1 exposure, contributes to the mutational profiles of two lemurs and is implicated in human hepatocellular carcinoma, particularly in Africa and Asia [6, 19, 37]. Studies with larger cohorts of lemurs in different geographical regions may provide valuable insights into the potential etiologies of hepatocellular carcinoma in these animals, and further research across primates may begin to illuminate the evolutionarily mediated differences in cancers across species.

## SUPPLEMENTARY DATA

Supplementary data is available at *EMPH* online.

## Supplementary Material

eoac016_Supplementary_Data
