# Supplementary figures and images for: Exome sequencing of hepatocellular carcinoma in lemurs identifies potential cancer drivers: A pilot study
Source: Evol Med Public Health. 2022 Apr 29;10(1):221–30. doi: 10.1093/emph/eoac016 (PMC9086584; doi:10.1093/emph/eoac016)

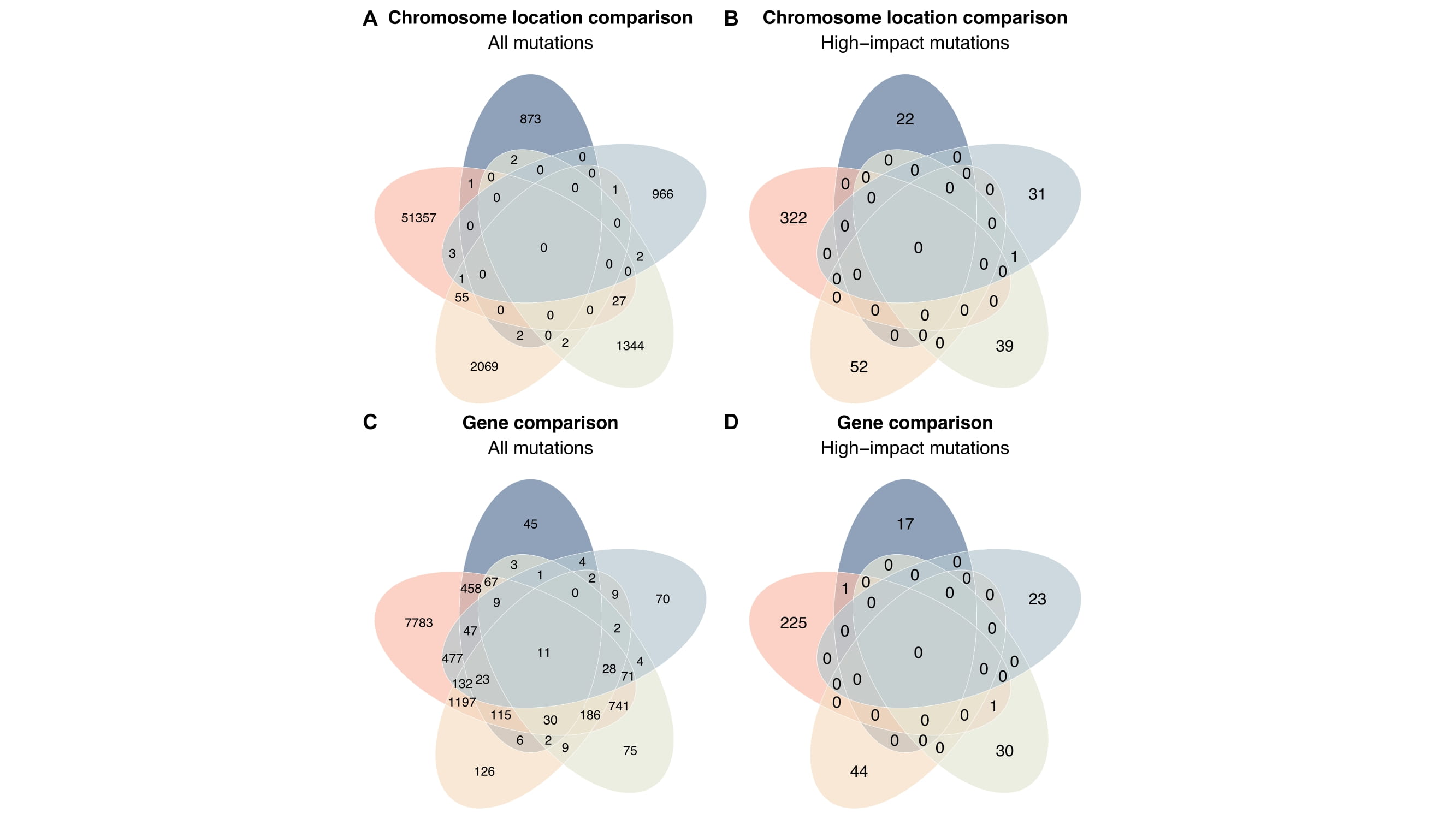

Supplement: eoac016_Supplementary_Data [file eoac016_supplementary_data.zip › Supplemental_Fig_2-1.jpg]

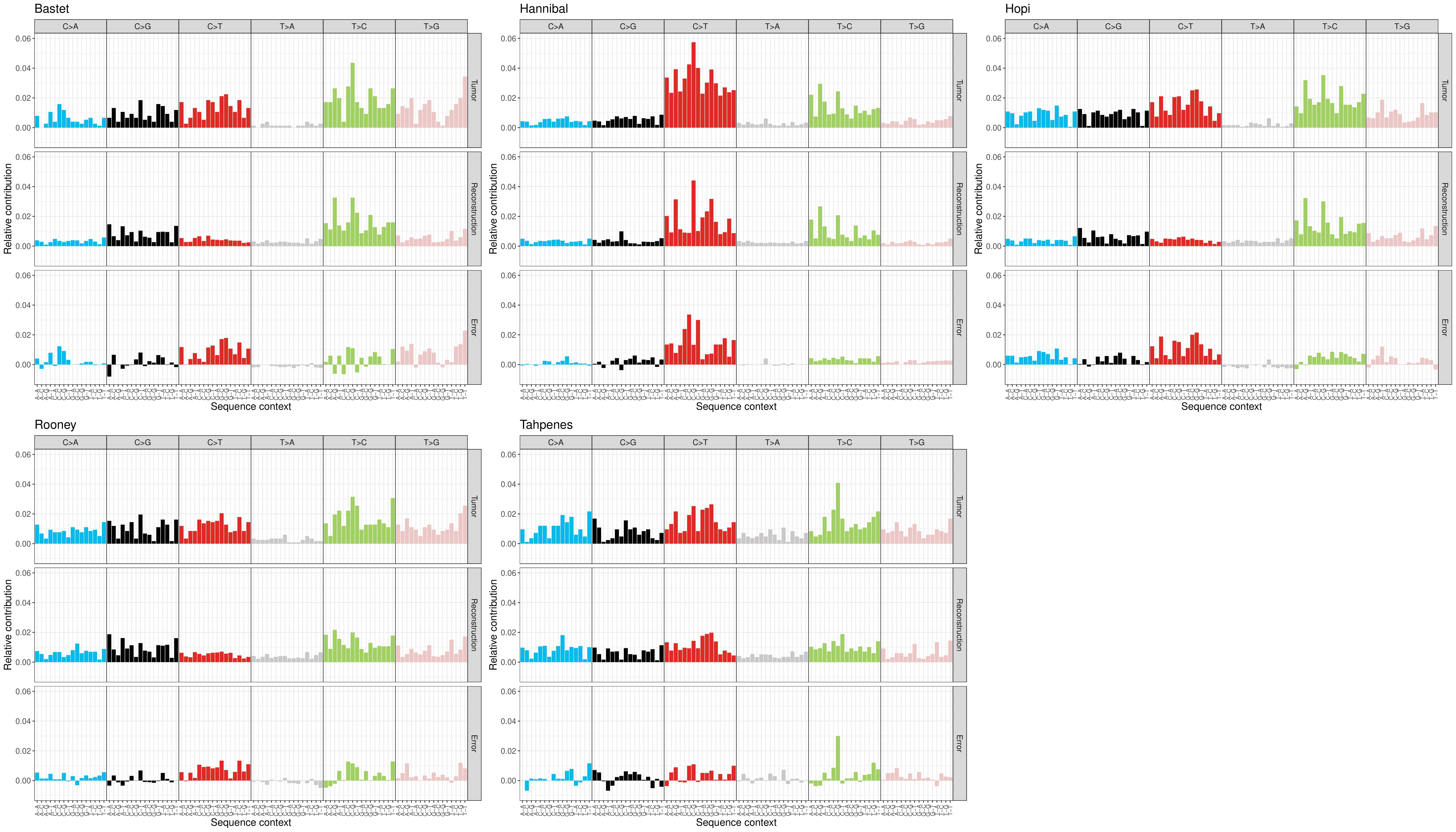

Supplement: eoac016_Supplementary_Data [file eoac016_supplementary_data.zip › Supplemental_Fig_3-1.jpg]

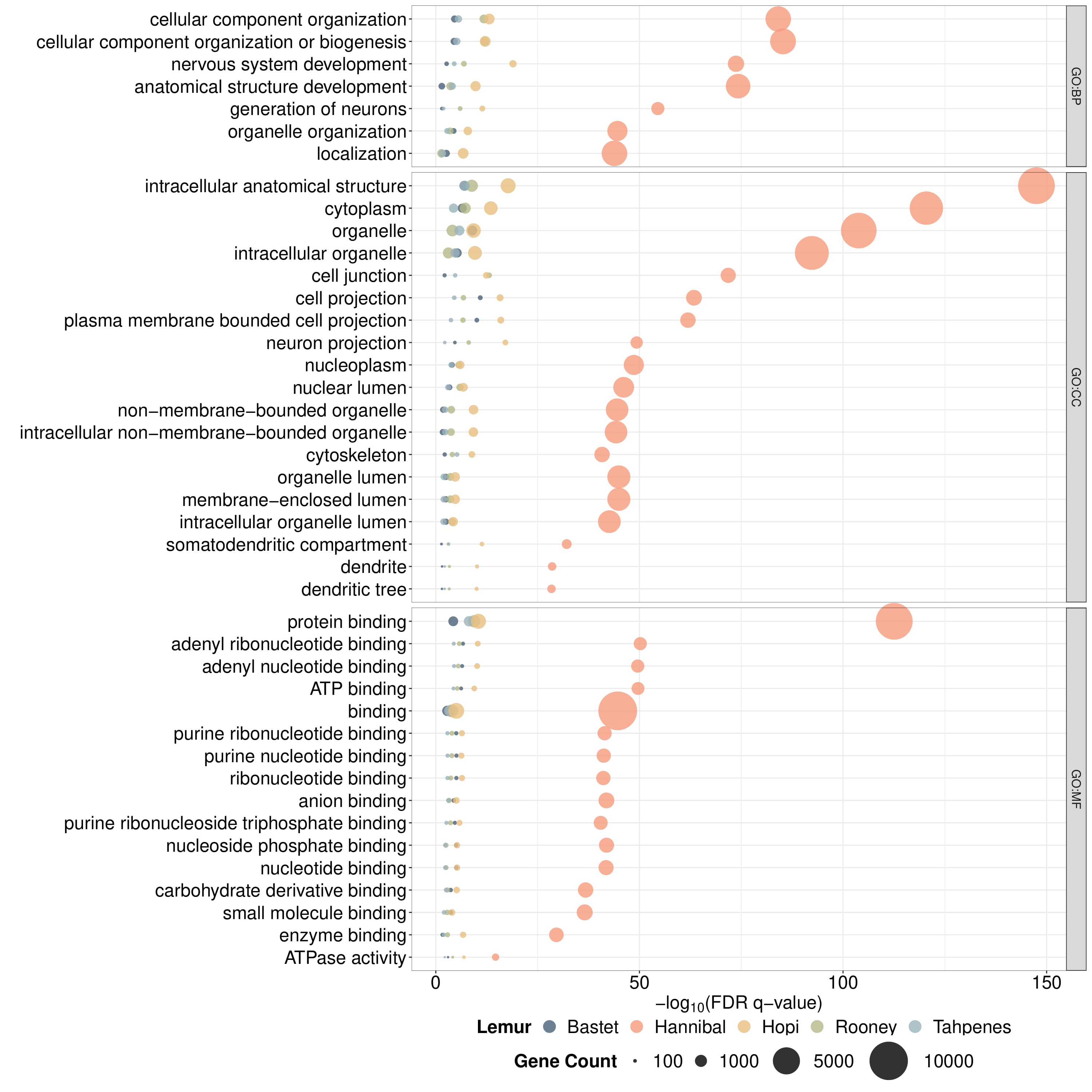

Supplement: eoac016_Supplementary_Data [file eoac016_supplementary_data.zip › Supplemental_Fig_4-1.jpg]

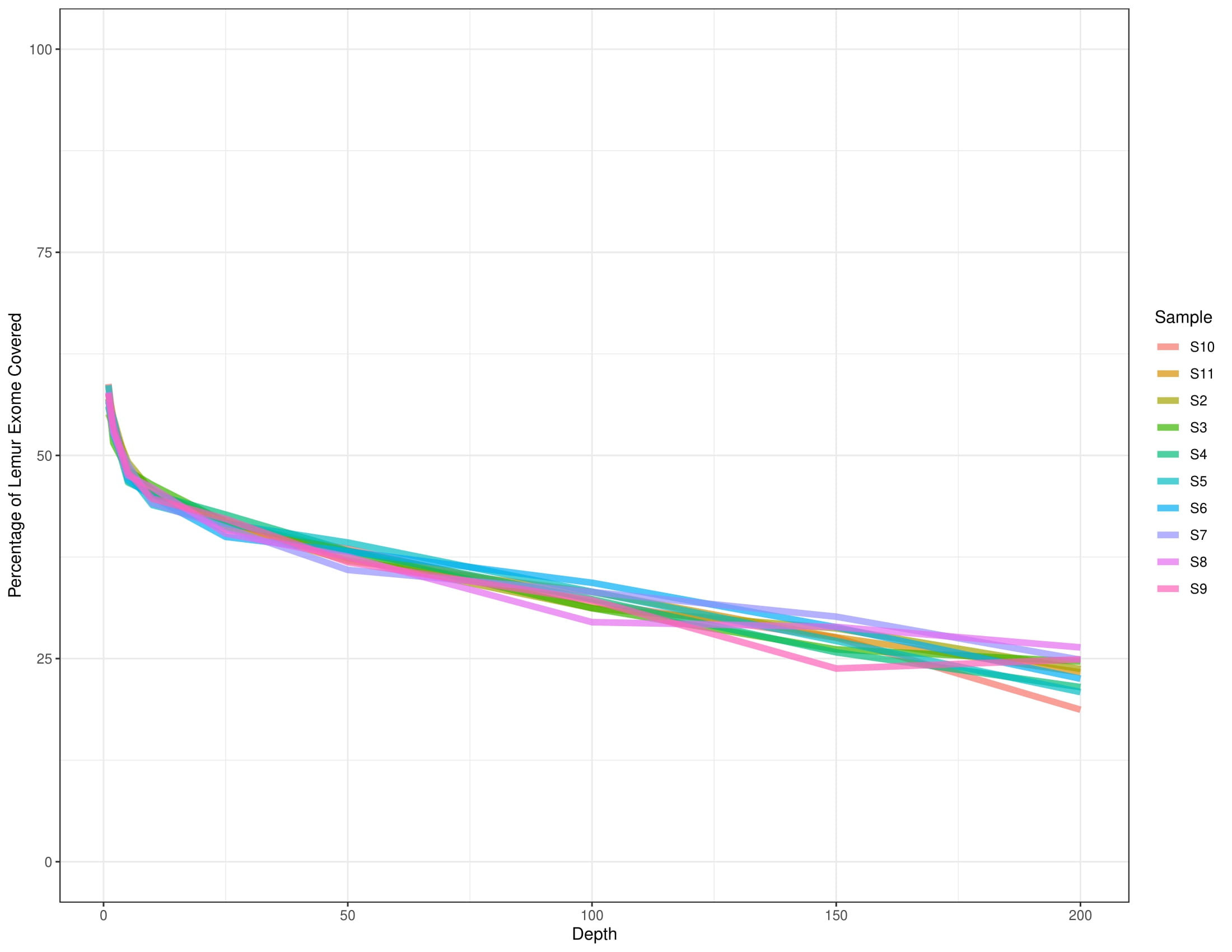

Supplement: eoac016_Supplementary_Data [file eoac016_supplementary_data.zip › Supplemental_Fig_1-1.jpg]
